# Development and validation of the regarding infection prevention and control among environmental service workers on knowledge, attitudes, practise, and experience questionnaire

**DOI:** 10.3389/fpubh.2022.1062199

**Published:** 2023-01-09

**Authors:** Xiaohang Chen, Pan Zhang, Ruhan Zhang, Shuting Li, Rui Cao, Fen Hu, Ying-Hui Jin, Likai Lin, Lin Cai, Bilong Feng, Chunhua Zhang, Xinghuan Wang

**Affiliations:** ^1^Institute of Hepatobiliary Diseases of Wuhan University, Zhongnan Hospital of Wuhan University, Wuhan, China; ^2^Department of Gastroenterology, Zhongnan Hospital of Wuhan University, Wuhan, China; ^3^Department of Critical Care Medicine, Zhongnan Hospital of Wuhan University, Wuhan, China; ^4^Department of Endocrinology, Zhongnan Hospital of Wuhan University, Wuhan, China; ^5^Department of Nursing, Zhongnan Hospital of Wuhan University, Wuhan, China; ^6^Hubei Engineering Center for Infectious Disease Prevention, Control, and Treatment, Wuhan, China; ^7^Center for Evidence-Based and Translational Medicine, Zhongnan Hospital of Wuhan University, Wuhan, China; ^8^Institute of Hospital Management, Wuhan University, Wuhan, China; ^9^Department of Orthopedics, Zhongnan Hospital of Wuhan University, Wuhan, China; ^10^Research Center of Wuhan for Infectious Diseases and Cancer, Chinese Academy of Medical Sciences, Wuhan, China; ^11^Department of Urology, Zhongnan Hospital of Wuhan University, Wuhan, China

**Keywords:** infection prevention and control, environmental service worker, knowledge, attitude, practise, experience, Delphi method

## Abstract

**Purpose:**

This study aimed to develop and test the validity and reliability of the Knowledge, Attitudes, Practise, and Experience regarding Infection Prevention and Control-associated Questionnaire for environmental service workers.

**Design:**

This study was a development and validation study of a questionnaire using multiple methods, including literature review, questionnaire survey, and Delphi technique.

**Methods:**

Phase I of the study entailed the development of items through an extensive literature review and two round Delphi process with 15 experts specialised in infection prevention and control, environmental service worker management, or scale construction to examine the content validity of the questionnaire. Phase II involved administering the questionnaire to a convenience sample of 1,176 environmental service workers from the public hospital from 13 provinces in China to evaluate its construct validity and reliability.

**Findings:**

In the two rounds of Delphi consultation, the recovery rate were 93.75 and 100%. Moreover, the expert authority coefficient was 0.93, and the coordination coefficients of expert opinions in the first round were as follows: correlation of 0.204 and importance of 0.249 for the first-level index; correlation of 0.128 and importance of 0.142 for the secondary index. In round two, the coordination coefficients of expert opinions were as follows: correlation of 0.221 and importance of 0.221 for the first-level indicators; correlation of 0.096 and importance of 0.101 for the secondary index. The results for the index were *P* < 0.05 for the two rounds. The pilot survey shows the instrument was excellent content validity (S-CVI/Ave = 0.989). The overall internal consistency was excellent (Cronbach's α = 0.967). The questionnaire ultimately comprised four first-level indices (knowledge, attitudes, practise, and experience) and 49 second-level indices.

**Conclusion:**

The Questionnaire demonstrated good reliability and validity and is effective in measuring levels of infection prevention and control-related knowledge, attitudes, practise, and experience among environmental service workers. It will provide a tool for future national investigations of the current infection prevention and control situation among environmental service workers. Future research should explore determinants of environmental service workers' knowledge, attitudes, practise, and experience and associations between infection prevention and control knowledge, attitudes, practises, and experience.

## 1. Introduction

Health care-acquired infections (HAIs)^1^ are a serious global public health issue, affecting millions of patients worldwide every year (1). The burden of HAIs needs to be highlighted not only because of the large number of patients affected every year but also for their significant impact in terms of excess costs, prolonged hospital stay-attributable mortality, and other complications (2). An estimated 15% of patients globally develop one or more infections during a hospital stay, with the greatest risk in low-income countries (3). In Europe alone, HAIs cause 16 million extra days of hospital stay and 37,000 attributable deaths and contribute to an additional 110,000, almost 9 million deaths are recorded every year (4). Pathogens linked with HAIs can not only cause disease but also survive in the hospital environment for weeks. More evidence suggests that the hospital environment should be cleaned and disinfected to prevent pathogen cross-transmission and remove infectious agents from fomites in the health care environment (5–7).

With the ongoing COVID-19 pandemic (8), strengthening infection prevention and control (IPC)^2^ was endorsed to reduce HAIs and prevent and control outbreaks by the World Health Organisation (WHO) (9).^3^ Over the last few years, the important role of hospital environment cleaning and disinfection in IPC has become more apparent (10). Numerous studies have shown that environmental service workers (ESWs)^4^ are a weak link in IPC in hospitals (11–13). Meanwhile, ESWs play a central role in effective environmental cleaning and disinfection, the critical frontline of defence against HAIs (14). In the context of the COVID-19 pandemic, we urgently need to understand the current situation of ESWs regarding their knowledge, attitudes, practise, and experience of IPC.

However, through a literature review, we found that the ESW assessment scale mostly addresses knowledge, attitudes, and practises of routine cleaning and disinfection of ESWs. The results showed that ESWs lacked knowledge of environmental disinfection (15, 16). There was no standardised instrument to evaluate IPC among ESWs. Therefore, it was important to develop a scientific method to develop a valid and reliable instrument for measuring ESWs' IPC practises considering knowledge, attitudes, practises, and experience. Considering that COVID-19 poses a challenge to IPC, we added COVID-19 to the questionnaire, for example, how to deal with the medical waste generated by COVID-19 patients and how to arrange the room disinfection of COVID-19 patients. This study aimed to develop a Knowledge, Attitudes, Practise, and Experience regarding Infection Prevention and Control-associated Questionnaire (KAPE-IPC-Q)^5^ for Chinese ESWs using the application of the Delphi technique and a pilot survey. The study findings will provide a tool for future national investigations of the current IPC situation among ESWs.

## 2. Methods

Email invitations were sent to experts from all over China to participate on 15th August 2021 to discuss the final questionnaire.

### 2.1. Scoping review and development of the first version of the item pool

Multidisciplinary teams were formed consisting of 12 members, including one methodological expert on tool building, one IPC manager, one research methodologist, two clinical medicine experts, three clinical nursing experts, and four IPC nurses.

A literature review, official documents, focus interviews, and team discussions selected the item pool for the questionnaire. (a) A literature review was conducted using the PubMed and ProQuest databases to define HAI, determine the risk factors and prevention measures, and establish a questionnaire item pool. (b) We searched websites, including the Chinese Centre for Disease Control (CDC) (6), Joint Commission International, WHO guidelines about IPC among health workers, and government documents (9, 17–20). (c) Focus interviews were conducted between the team members and 16 ESWs and their eight leaders. (d) Discussions among all team members after the literature review and the focus interviews were conducted; the team members classified the literature, policies, and interview information and removed the duplicate content to establish an item pool.

### 2.2. Delphi process

**Figure 1:**
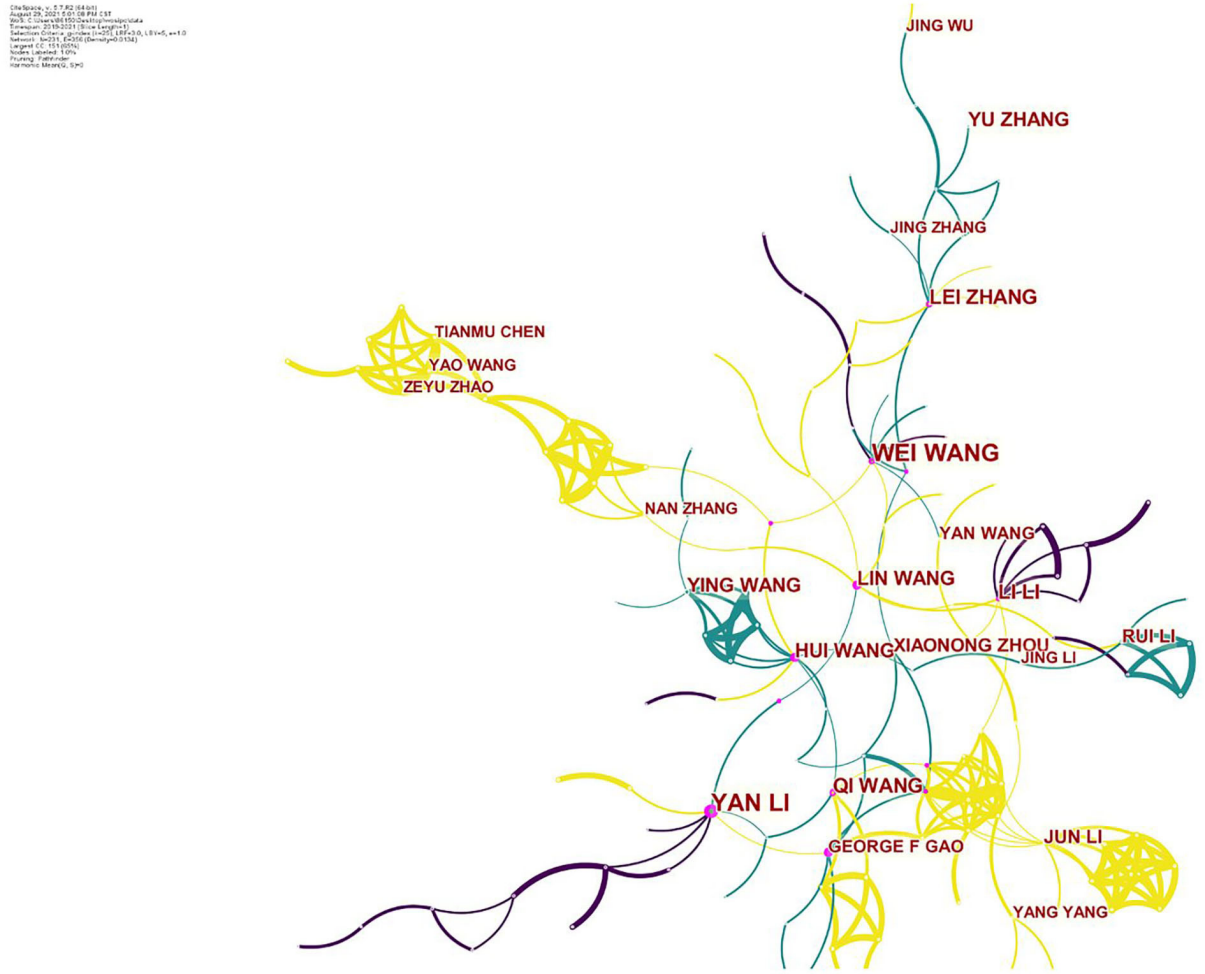
Expert selection.

To ensure broad and varied expertise in the field of IPC and ESW management, CiteSpace V5.7.R2 was used to select potential experts (Figure 1). The potential experts were chosen by discussing and consulting the research field of interest over the past 3 years, and the experts building the panel are composed of the following characteristics: (1) own experience and expertise in IPC, ESWs management in a scientific or clinical context, scale development, or research personnel, (2) at least 10 years of relevant experience, (3) possession of a senior professional post, and (4) broad geographic spread (Table 1).

**Table 1:** Expert information.

**Project**	**Delphi round 1**		**Delphi round 2**	
	**Frequency**	**Percentage (%)**	**Frequency**	**Percentage (%)**
**Sex**				
Male	2	12.5%	1	6.67%
Female	14	87.5%	14	93.33%
**Age**				
31–40 y	3	18.75%	2	13.33%
41–50 y	8	50%	8	53.33%
≥51 y	5	31.25%	5	33.33%
**Degree**				
Undergraduate	7	43.75%	7	46.67%
Master's	6	37.5%	5	33.33%
Ph.D.	3	18.75%	3	20%
**Years of working**				
11–20 y	5	31.25%	4	26.67%
21–30 y	5	31.25%	5	33.33%
31–40 y	6	37.5%	6	40%
**Professional title**				
Senior vice title	7	43.75%	6	40%
Senior title	9	56.25%	9	60%
**Nature of work**				
Hospital infection prevention and control personnel	5	31.25%	4	26.67%
Clinical staff	5	31.25%	5	33.33%
Managers	4	25%	4	26.67%
Scale development	1	6.25%	1	6.67%
Researcher	1	6.25%	1	6.67%

Email invitations were sent to 16 experts from all over China to participate (from 10 major cities, including Beijing, Shanghai, Guangdong, Hubei, Jiangsu, Jiangxi, Sichuan, Hebei, Liaoning, and Shanxi). Upon acceptance, experts were included in the Delphi process to generate consensus but did not know the identity of the other participants. Participation was voluntary, and consent was implied if the participant responded to the survey. The Delphi questionnaire comprised two sections. The first section included the complete KAPE-IPC-Q with detailed descriptions of each subscale and item. Experts were asked to rate each item on a five-point Likert scale (1 = not important/not correlated, 5 = very important/correlated) with additional blanks to allow them to fill in revision comments. At the end of the questionnaire, experts could also provide opinions or suggestions for content that had not been included in the questionnaire. The second section asked for experts' personal information (i.e., age, professional title, occupation, and education level).

### 2.3. Pilot survey

The pilot survey was performed with a convenience sample of ESWs at the public hospital from 13 provinces in China. The inclusion criteria were ESWs employed by hospitals. They were asked to provide their comments about problems in completing the questionnaire, including whether it was clear and understandable and whether the content was relevant to their practise, and we recorded the time spent filling out the questionnaire.

**Figure 2:**
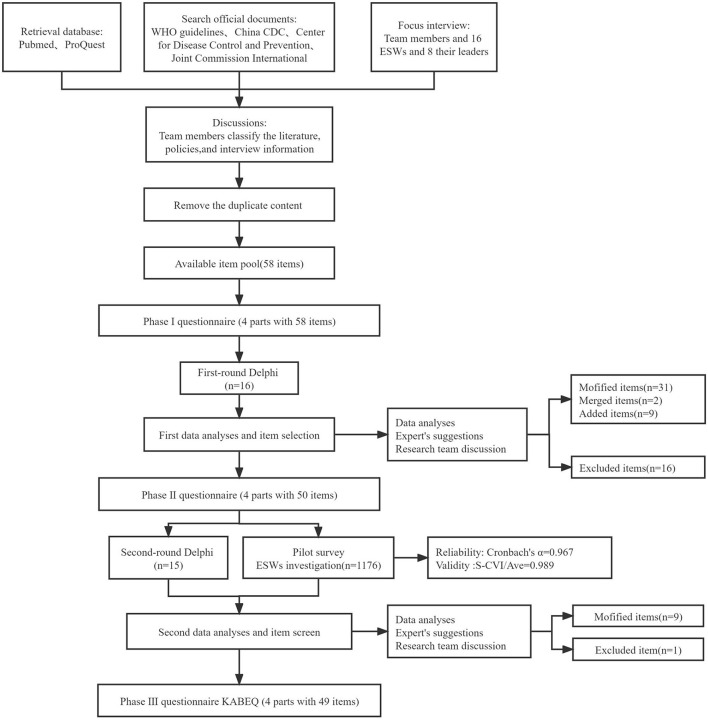
Flow chart of the development questionnaire.

The questionnaire comprised two sections. The first section assessed sociodemographic variables (i.e., age, education level, marital status, and income), the second section comprised KAPE-IPC-Q items. Each item was rated on a three—or five-point Likert scale, and there were eight reverse scoring questions in this questionnaire, with higher total scores representing greater knowledge, attitudes, practise, and experience regarding IPC. Given that the KAPE-IPC-Q is self-reported, ESWs who could read and write completed the questionnaire by themselves; however, ESWs who struggled with reading and writing received the interviewer-administered survey, and their responses were recorded by the interviewer verbatim (Figure 2).

### 2.4. Data analyses

#### 2.4.1. Delphi technique data

In each Delphi round, experts were asked to rate each statement according to its importance and correlation to the questionnaire using a five-point Likert scale. Moreover, they were invited to comment on each item. Questionnaire item data are presented as the mean, standard deviation (SD), coefficient of variation (CV), and Kendall *W*. The larger the mean, the more important the index, and vice versa; the larger the CV, the greater the difference in the experts' understanding of the content of the questionnaire, and the lower the consistency of their opinions. Kendall's *W*-test was used to confirm the relevance of the experts' responses for all items (21). The higher the Kendall *W*-value, the higher the level of agreement among the members of the expert panel. The authority coefficient (Cr) is used to evaluate the degree of authority of experts, which is related to the technical ability of evaluation indicators and is determined by the familiarity and the mean value of the judgement basis (22).

According to the results of the Delphi process, the item importance average score ≥ 3.5 points or the coefficient of variation ≤ 0.25 was used as the screening criteria (23), and the items were screened after the experts' revision opinions and reviewed after repeated discussions among the research team members.

#### 2.4.2. Pilot survey data

Using these data, we performed further item selection and revision through analyses of the internal consistency (Cronbach's α), and a Cronbach's α of ≥0.70 was considered to indicate acceptable reliability (24). Content validity reflects the consistency between the content measured by the questionnaire and the content to be measured. The S-CVI/Ave value is used to express content validity. At present, the most commonly used evaluation index of content validity is the S-CVI/Ave. When the S-CVI/Ave > 0.90, the content validity of the questionnaire is good (25).

A database was constructed using EpiData version 3.5.2 and all data were double-entered by two data managers to avoid any possible data entry errors. Statistical analyses were performed using SPSS software (version 23.0; SPSS Inc.). A *p*-value < 0.05 (two-tailed) was considered significant.

## 3. Results

### 3.1. Item pool

The initial item pool comprised 58 items describing knowledge, attitudes, practise, and experience regarding IPC. The items were divided into hospital environmental surface cleaning and disinfection management, isolation systems and hand hygiene, occupational protection, medical treatment, and COVID-19-related terminal disinfection.

### 3.2. First-round Delphi process

Sixteen eligible experts were recruited in the first round of the Delphi method and 15 experts provided effective responses. Sixteen items were excluded, 31 items were modified, two items were merged, and nine items were added because their CVs were >0.25 or based on expert opinions (Table 2). The remaining 33 items all had CVs ≤ 0.25 [correlation value Kendall's *W* = 0.204/0.128; importance value Kendall's *W* = 0.249/0.142, *p* < 0.001 (Table 3)].

**Table 2:** Results of Delphi round 1 (*N* = 16).

**Abbreviated item content of the KABEQ**	**Correlation value**		**Importance value**	
	$\bar{X}$ ±**S**	**CV**	$\bar{X}$ ±**S**	**CV**
1. Knowledge	4.733 ± 0.458	0.097	4.8 ± 0.414	0.086
a1	4.933 ± 0.258	0.052	4.933 ± 0.258	0.052
a2	4.733 ± 0.799	0.169	4.8 ± 0.775	0.161
a3	4.867 ± 0.516	0.106	4.867 ± 0.516	0.106
a4	4.667 ± 0.617	0.132	4.733 ± 0.594	0.125
a5	4.933 ± 0.258	0.052	5 ± 0	0
a6	4.8 ± 0.414	0.086	4.733 ± 0.594	0.125
a7	5 ± 0	0	5 ± 0	0
a8	5 ± 0	0	5 ± 0	0
a9	4.867 ± 0.352	0.072	4.933 ± 0.258	0.052
a10	4.867 ± 0.516	0.106	4.933 ± 0.258	0.052
a11	4.733 ± 0.594	0.125	4.8 ± 0.414	0.086
a12	4.933 ± 0.258	0.052	4.933 ± 0.258	0.052
a13	4.8 ± 0.561	0.117	4.8 ± 0.561	0.117
a14	4.8 ± 0.561	0.117	4.733 ± 0.799	0.169
a15	5 ± 0	0	5 ± 0	0
a16	4.8 ± 0.414	0.086	4.8 ± 0.414	0.086
a17	4.933 ± 0.258	0.052	5 ± 0	0
a18	5 ± 0	0	5 ± 0	0
a19	4.867 ± 0.516	0.106	4.867 ± 0.516	0.106
a20	4.933 ± 0.258	0.052	4.933 ± 0.258	0.052
a21	4.867 ± 0.352	0.072	4.933 ± 0.258	0.052
2. Attitude	4.933 ± 0.258	0.052	5 ± 0	0
b1	5 ± 0	0	5 ± 0	0
b2	4.733 ± 0.458	0.097	4.867 ± 0.352	0.072
b3	4.933 ± 0.258	0.052	5 ± 0	0
b4	4.6 ± 0.632	0.137	4.667 ± 0.488	0.105
b5	4.267 ± 1.1	0.258	4.733 ± 0.458	0.097
b6	4.667 ± 0.617	0.132	4.8 ± 0.414	0.086
3. Practise	4.933 ± 0.258	0.052	5 ± 0	0
c1	4.667 ± 0.724	0.155	4.8 ± 0.414	0.086
c2	4.6 ± 0.632	0.137	4.867 ± 0.352	0.072
c3	4.867 ± 0.352	0.072	4.933 ± 0.258	0.052
c4	4.867 ± 0.352	0.072	5 ± 0	0
c5	4.8 ± 0.561	0.117	4.867 ± 0.516	0.106
c6	4.867 ± 0.64	0.131	4.8 ± 0.561	0.117
c7	4.667 ± 0.724	0.155	4.667 ± 0.724	0.155
c8	4.533 ± 1.125	0.248	4.667 ± 0.9	0.193
c9	4.667 ± 0.816	0.175	4.8 ± 0.561	0.117
c10	4.8 ± 0.561	0.117	4.8 ± 0.561	0.117
c11	4.933 ± 0.258	0.052	5 ± 0	0
c12	4.333 ± 1.175	0.271	4.4 ± 1.121	0.255
c13	4.867 ± 0.352	0.072	4.933 ± 0.258	0.052
c14	4.933 ± 0.258	0.052	4.8 ± 0.775	0.161
c15	4.933 ± 0.258	0.052	4.933 ± 0.258	0.052
c16	4.867 ± 0.352	0.072	4.867 ± 0.352	0.072
c17	4.867 ± 0.352	0.072	4.867 ± 0.352	0.072
c18	4.933 ± 0.258	0.052	4.933 ± 0.258	0.052
c19	4.667 ± 0.816	0.175	4.667 ± 0.816	0.175
c20	4.8 ± 0.414	0.086	4.867 ± 0.352	0.072
c21	5 ± 0	0	5 ± 0	0
c22	4.933 ± 0.258	0.052	4.933 ± 0.258	0.052
c23	4.867 ± 0.352	0.072	4.867 ± 0.352	0.072
c24	4.933 ± 0.258	0.052	4.933 ± 0.258	0.052
4. Experience	4.6 ± 0.632	0.137	4.6 ± 0.632	0.137
d1	4.867 ± 0.352	0.072	4.8 ± 0.414	0.086
d2	4.867 ± 0.352	0.072	4.933 ± 0.258	0.052
d3	4.4 ± 0.737	0.167	4.333 ± 0.724	0.167
d4	4.533 ± 0.64	0.141	4.533 ± 0.64	0.141
d5	4.6 ± 0.507	0.11	4.6 ± 0.507	0.11
d6	4.8 ± 0.414	0.086	4.8 ± 0.414	0.086
d7	4.6 ± 0.507	0.11	4.6 ± 0.507	0.11

**Table 3:** Coordinate coefficients of expert advice for two rounds of consultations.

	**Hierarchical level**	**Index (*n*)**	**Correlation value**			**Importance value**		
			**Kendall's** ***W***	***X** ^2^*	***P***	**Kendall's W**	***X** ^2^*	***P***
Round 1	First-level	4	0.204	9.171	0.027	0.249	11.195	0.011
	Second-level	58	0.128	109.095	<0.001	0.142	121.668	<0.001
Round 2	First-level	4	0.221	9.923	0.019	0.221	9.923	0.019
	Second-level	50	0.096	70.44	0.024	0.101	74.035	0.012

### 3.3. Second-round Delphi process

In the second-round Delphi method, all 15 experts who responded to the first round returned suitable responses. All items had CVs ≤ 0.25, and one item was excluded, with nine modified items, based on expert opinions (Table 4). Some items in the questionnaire were not colloquial enough [correlation value Kendall's *W* = 0.221/0.096; importance value Kendall's *W* = 0.221/0.101, *p* < 0.05 (Table 3)].

**Table 4:** Results of Delphi round 2 (*N* = 15).

**Abbreviated item content of the KABEQ**	**Correlation value**		**Importance value**	
	$\bar{X}$ ±**S**	**CV**	$\bar{X}$ ±**S**	**CV**
1. Knowledge	5 ± 0	0	5 ± 0	0
a1	4.867 ± 0.352	0.072	5 ± 0	0
a2	4.800 ± 0.414	0.086	4.8 ± 0.414	0.086
a3	4.867 ± 0.352	0.072	4.933 ± 0.258	0.052
a4	4.8 ± 0.414	0.086	4.933 ± 0.258	0.052
a5	5 ± 0	0	5 ± 0	0
a6	4.867 ± 0.352	0.072	4.8 ± 0.414	0.086
a7	4.867 ± 0.352	0.072	5 ± 0	0
a8	4.867 ± 0.352	0.072	4.933 ± 0.258	0.052
a9	4.733 ± 0.458	0.097	4.867 ± 0.352	0.072
a10	4.867 ± 0.352	0.072	4.867 ± 0.352	0.072
a11	4.667 ± 0.488	0.105	4.8 ± 0.561	0.117
a12	4.933 ± 0.258	0.052	5 ± 0	0
a13	5 ± 0	0	5 ± 0	0
a14	4.8 ± 0.414	0.086	4.933 ± 0.258	0.052
a15	4.8 ± 0.414	0.086	4.933 ± 0.258	0.052
a16	4.933 ± 0.258	0.052	4.933 ± 0.258	0.052
2. Attitude	4.933 ± 0.258	0.052	4.933 ± 0.258	0.052
b1	4.8 ± 0.414	0.086	4.867 ± 0.352	0.072
b2	4.933 ± 0.258	0.052	4.933 ± 0.258	0.052
b3	4.933 ± 0.258	0.052	4.933 ± 0.258	0.052
b4	4.933 ± 0.258	0.052	4.867 ± 0.352	0.072
b5	4.867 ± 0.352	0.072	4.867 ± 0.352	0.072
b6	4.8 ± 0.414	0.086	4.867 ± 0.352	0.072
b7	4.867 ± 0.352	0.072	4.867 ± 0.352	0.072
3. Practise	5 ± 0	0	5 ± 0	0
c1	4.867 ± 0.352	0.072	4.933 ± 0.258	0.052
c2	4.867 ± 0.516	0.106	4.867 ± 0.516	0.106
c3	4.867 ± 0.352	0.072	4.933 ± 0.258	0.052
c4	5 ± 0	0	5 ± 0	0
c5	4.867 ± 0.352	0.072	4.867 ± 0.516	0.106
c6	4.867 ± 0.352	0.072	4.8 ± 0.414	0.086
c7	4.933 ± 0.258	0.052	5 ± 0	0
c8	4.933 ± 0.258	0.052	4.933 ± 0.258	0.052
c9	4.933 ± 0.258	0.052	4.933 ± 0.258	0.052
c10	4.933 ± 0.258	0.052	4.933 ± 0.258	0.052
c11	4.933 ± 0.258	0.052	4.933 ± 0.258	0.052
c12	5 ± 0	0	5 ± 0	0
c13	4.933 ± 0.258	0.052	4.933 ± 0.258	0.052
c14	4.867 ± 0.352	0.072	4.933 ± 0.258	0.052
c15	5 ± 0	0	4.933 ± 0.258	0.052
c16	4.867 ± 0.352	0.072	4.867 ± 0.352	0.072
c17	5 ± 0	0	5 ± 0	0
4. Experience	4.733 ± 0.458	0.097	4.733 ± 0.458	0.097
d1	4.867 ± 0.352	0.072	5 ± 0	0
d2	4.867 ± 0.352	0.072	4.867 ± 0.352	0.072
d3	4.933 ± 0.258	0.052	5 ± 0	0
d4	4.933 ± 0.258	0.052	5 ± 0	0
d5	4.667 ± 0.617	0.132	4.6 ± 0.632	0.137
d6	4.8 ± 0.561	0.117	4.867 ± 0.352	0.072
d7	4.933 ± 0.258	0.052	4.933 ± 0.258	0.052
d8	4.933 ± 0.258	0.052	4.867 ± 0.352	0.072
d9	4.533 ± 0.834	0.184	4.667 ± 0.816	0.175
d10	4.467 ± 0.743	0.166	4.467 ± 0.834	0.187

After two rounds of the Delphi and in-depth discussion among the team members, 18 items were excluded, 31 items were modified, two items were merged, and 10 items were added based on the exclusion criteria and expert opinions. The KAPE-IPC-Q ultimately comprised four first-level indices (knowledge, attitudes, practise, and experience) and 49 second-level indices (Table 5).

**Table 5:** Final tool.

**First-level indices**	**Second-level indices**
1. Knowledge	1.1. The working area of the hospital is divided into contaminated areas, potentially contaminated areas, and clean areas
	1.2. Tools and articles used in the ward cannot be brought into the restaurant
	1.3. Do not use the same cloth to wipe all bed units
	1.4. The concentration of chlorine-containing disinfectant for wiping and mopping the floor in the general ward is 500 mg/L
	1.5. You can wear a pair of gloves to wipe all bed units, and there is no need to disinfect hands between different bed units (reverse)
	1.6. Wash your hands after taking off your gloves
	1.7. When washing hands, rub your hands for at least 15 s
	1.8. The inside and outside of the mask can be directly touched by the hand (reverse)
	1.9. Wash hands immediately and replace gloves after they are torn
	1.10. Don not fill the bag with medical waste
	1.11. Medical waste can be collected without gloves (reverse)
	1.12. The hospital bed can be cleaned without disinfection after the patient is discharged (reverse)
	1.13. Domestic waste and medical waste cannot be mixed
	1.14. Patients with COVID-19 who are suspected or diagnosed should wear a medical respirator (such as N95 mask / KN95 mask).
	1.15. The waste products of COVID-19 patients are recycled into black garbage bags (reverse).
2. Attitude	2.1. I think cleaning can ensure the safety of the hospital environment
	2.2. I think it is important to perform hand hygiene
	2.3. I think it is very important to prepare disinfectants according to the requirements
	2.4. I think it is important to finish the work according to the working standards of the hospital and cleaning company
	2.5. I think it is important to wear a mask, gloves, and isolation gown if necessary
	2.6. I think it is important to monitor my health every day and report illness on time
	2.7. I think it is important to receive training according to the requirements of the hospital and cleaning company
3. Practise	3.1. When I see a special infection sign in the room or beside the bed, I will wear personal protective equipment before starting work as needed
	3.2. When the ground is polluted by a large amount of blood and body fluids of patients, I will first sweep it with a broom and then mop it with a mop
	3.3. When cleaning in the office and ward, I will use rags of different colours.
	3.4. When I clean the COVID-19 patient's ward, I usually set the concentration of chlorine disinfectant to 500 mg/L (reverse)
	3.5. After I prepare the disinfectant, I will use disinfectant concentration test tools (such as professional chlorine test paper) to test whether the disinfectant formulation is appropriate.
	3.6. I will wash my hands before I wear a mask
	3.7. When I come into contact with the patient's body fluids/blood or secretions, I will wash my hands with flowing water first, and then disinfect my hands
	3.8. I will wash my hands when I touch the door handle, elevator button, and department phone in the ward
	3.9. When the mask is contaminated or wet, I will replace it immediately
	3.10. When I wear a mask, I put one end of the metal strip on top, put it on the bridge of my nose, and then clamp the nose clip
	3.11. When cleaning the ward, if I am stabbed by a sharp weapon, I will squeeze the wound immediately, clean and disinfect it, and then report to the supervisor
	3.12. In case of fever, cough, fatigue, and other symptoms, I will immediately report to the supervisor and go to the fever clinic
	3.13. When dealing with COVID-19 patients, I will use a single yellow trash bag to pack the medical waste (reverse)
	3.14. I will mark when I deal with medical waste from suspected and confirmed COVID-19 patients.
	3.15. I will tie the bag tightly with a tie when I deal with medical waste from the suspected and confirmed COVID-19 patients.
	3.16. During the COVID-19 pandemic, I will submit itinerary/health codes daily.
	3.17. I will squeeze the bag with my hand when I collect disposable isolation clothes, protective clothing, and other items that are about to overflow (reverse)
4. Experience	4.1. I can receive regular training related to cleaning work
	4.2. The training I received will enable me to carry out my work smoothly
	4.3. I can have all the necessary cleaning tools at work (such as dishcloths and disinfectant)
	4.4. I can get enough personal protective equipment (such as masks and gloves)
	4.5. I can get other people's attention in my work
	4.6. I have participated in the physical examination organised by the unit (except regular nucleic acid testing)
	4.7. The hospital or company will cheque my work
	4.8. The hospital or company will seek my opinions or suggestions
	4.9. I can handle the current workload
	4.10. I am satisfied with my income (salary, bonus, etc.)

#### 3.3.1. Pilot survey

We used the collected questionnaires for the pilot survey, 1,176 ESWs participated. The Cronbach's α coefficient was 0.967, and the S-CVI/Ave was 0.989. Therefore, the definitive version of the KAPE-IPC-Q after the two rounds of testing had four sections with 49 items.

## 4. Discussion

IPC management is not only the focus of hospital management but also an important public health problem (26). ESWs in medical institutions are vital for the development of IPC, and health authorities must develop effective strategies to improve IPC compliance of ESWs. To formulate effective measures, it is essential to obtain the current situation on knowledge, attitudes, practise, and experience among ESWs regarding IPC. Since there was no available international measurement tool at the time of our survey, we designed a questionnaire entitled knowledge, attitudes, practise, and experience among ESWs regarding IPC in Chinese hospitals and examined its reliability and validity. We believe that our study has filled an important gap in the assessment of knowledge, attitudes, practise, and experience regarding IPC for ESWs in developing countries (e.g., China).

First, we based on information from the China Centres for Disease Control and Prevention (CDC), Joint Commission International, and WHO. Most of these websites have a strong global influence, especially in terms of hospital IPC. And according to the literature review and theoretical framework related to hospital environmental surface cleaning and disinfection management, the isolation system and hand hygiene, occupational protection, medical treatment, and COVID-19-related terminal disinfection. And then we conducted several research group meetings and two rounds of the Delphi process, combining the theoretical framework of IPC for ESWs. Accordingly, this study developed a questionnaire on knowledge, attitudes, practise, and experience regarding IPC among ESWs, it is scientific, comprehensive, and practical.

In the Delphi method, the number of experts in the panel was functionally related to the accuracy of the study results, which suggests that at least 10–18 expert members per panel are required to achieve a range of opinions (27). The quality of the consulting experts directly determines the success or failure of the Delphi method (28). In this study, 15 experts had influence and outstanding performance in IPC, scale development, evidence-based medicine, and hospital management, all with rich work experience and high-level job titles and academic qualifications, thus ensuring that the indicator system was rich in practical experience and a theoretical basis; the experts came from 10 different provinces (regions). Therefore, using the Delphi method, the quality of experts was high, and the quality index system was more reliable. Furthermore, this questionnaire had good reliability and validity through a pilot survey. Accordingly, the questionnaire is reliable.

The questionnaire items were set up as colloquially as possible because the respondents were culturally diverse and had a wide distribution of ages. Because of extensive outsourcing of hospital cleaning services, high turnover and inadequate training occur around the world (29). The inevitable result: A study of more than 1,000 patient rooms across 23 hospitals revealed that <50% of surfaces are properly cleaned (30). Around the world, ESWs' current situation is similar to that in China (31). Although our framework was developed in the Chinese context, we believe that it can be used in other countries for assessing knowledge, attitudes, practise, and experience regarding IPC for ESWs. Accordingly, the questionnaire is practical.

Knowledge and information are the foundation and the establishment of positive, correct beliefs and attitudes. Beliefs and attitudes are the motivating force for healthy behaviour change. Moreover, these two factors enhance and help evaluate IPC knowledge in ESWs as well as help ESWs recognise possible interventions to improve behavioural and attitudinal changes. Positive attitudes and behavioural changes are driven by the level of knowledge and perceptions towards preventive practices (32).

Previous studies have showed that surface contamination with pathogens results from inadequate cleaning by ESWs (11–13). Other studies have shown that the important measure to reduce the risk of hospital infection was correct and effective cleaning and disinfection (33). With this in mind, the knowledge and behavioural dimensions of this questionnaire include a large amount of hospital environmental surface cleaning and disinfection management, content, such as “Ordinary wiping and floor mopping disinfectant formulation: 5,000 ml (5 L) water + five tablets of chlorine-containing effervescent tablets”; the experts suggested changing to a specific concentration of chlorine-containing disinfectant, which was later changed to “the concentration of chlorine disinfectant in the common ward wipes and wet mops on the ground is 500 mg/L.” and “I can use chlorine disinfectants (e.g., 84 disinfectant) test tools to detect whether the disinfectant formulation is appropriate.” The experts recommended changing the chlorine disinfectant test tool to professional chlorine test paper when cleaners formulate the disinfectant; they used the disinfectant concentration test tool (such as professional chlorine test paper) to test whether the disinfectant formulation was appropriate. Cleaning and disinfection of the surface of the hospital environment are very important for cleaners.

Respondents reported that hand hygiene knowledge and practise among ESWs were unsatisfactory, but hand hygiene for ESWs may contribute to reducing the risk of cross-transmission (34). This questionnaire contained 8 items to investigate hand hygiene in ESWs. Hand hygiene is a complex behaviour that is easily influenced by knowledge, attitudes, values, and beliefs (35). Therefore, a survey of the knowledge and behaviour of hand hygiene among cleaners can facilitate the development of hand hygiene training in later stages.

When cleaning staff clean debris and sharps, they are likely to be stabbed by infusion needles, injection needles, scissor knives, or other medical devices (36). Most of the cleaners in China have not been trained in formal medical knowledge, and their awareness of their protection in daily operations is relatively weak. The treatment after being stabbed by needles is not standardised enough, and even a small number of cleaners have not undergone any treatment. The reason cleaners may not report a needlestick injury to the hospital infection office is that the reporting procedure is cumbersome and deemed unnecessary. There are still considerable safety hazards in the treatment of needle puncture injuries of cleaning staff, and the management mechanism of acupuncture wounds in hospitals also needs to be improved. Based on the above situation, this questionnaire set up the item “when cleaning the ward and pricked by a sharp weapon, I will immediately squeeze, clean, and disinfect and then report to my superior”; on the one hand, the handling of cleaning staff's needle puncture wound would be investigated, and on the other hand, the cleaner would be informed on how to correctly handle the needle puncture injury process.

One of the main risk factors that threaten a patient's health is HAIs (37). There are nearly 20 million HAI patients in the United States each year, of which nearly 90,000 die, and the direct economic loss is ~$28 billion to $45 billion (38). China surveyed 1,766 hospitals in 2014 and found that the HAI prevalence rate was 2.67%, which severely affected the patients' prognoses and increased the financial burden on patients, but 20–30% of HAIs were preventable (39). Central to effective disinfection is the ESW (40). When a hospitalised patient suffers an infection, the next patient to occupy their room has a 6-fold greater risk of acquiring the same pathogen (41). Terminal disinfection is an effective method of removing bacteria from the ward or bed (42). The questionnaire designed by the study set up the item “the bed unit after the patient is discharged from the hospital cannot be cleaned and disinfected” to understand the mastery of the terminal disinfection knowledge of the cleaning staff in preparation for future targeted hospital IPC training.

At present, the novel coronavirus is spreading rapidly around the world, the pressure to prevent the transmission of the virus continues to increase worldwide, and the task of epidemic prevention and control is still arduous (43). ESWs play an important role in the prevention of HAIs (16). The questionnaire designed for this study included the item “The use of a medical protective mask (e.g., N95 mask/KN95 mask) when exposed to suspected or confirmed COVID-19” and how to deal with medical waste generated by COVID-19 patients. The item “When I have symptoms such as fever, cough, and fatigue, I will immediately report it to my superior manager and go to the fever clinic,” which helps the cleaners to do a good job in epidemic prevention and control during the novel coronavirus epidemic.

A strength of this study was that the questionnaire was designed to fit a daily work scenario, which was easier for ESWs to understand. To minimise the misunderstanding of the medical terms included in the survey, the questionnaire avoided using medical jargon where possible and completed piloting by ESWs. It is recommended that a response rate of 70% be achieved for each round of the Delphi method to reduce bias and reach a meaningful consensus (44). Accordingly, the present study had response rates of over 70% between the rounds. It was considered that two rounds were sufficient to reach a consensus, and the consequence of undertaking further rounds included participant fatigue and higher drop-out rates (45).

This study has some limitations. First, as this study was conducted only in China, further validation studies are necessary for generalisation to other countries. Furthermore, the Delphi technique has been criticised for a lack of standardisation, and the reliability of the Delphi technique has not been confirmed (44–47).

## 5. Conclusion

In summary, this study was based on the Delphi method to develop a questionnaire on knowledge, attitudes, practise, and experience regarding IPC among ESWs that has good reliability and validity and has certain scientific and practical value.

## Data availability statement

The original contributions presented in the study are included in the article, further inquiries can be directed to the corresponding authors.

## Ethics statement

The studies involving human participants were reviewed and approved by Zhongnan Hospital of Wuhan University Medical Ethics Committee. The patients/participants provided their written informed consent to participate in this study.

## Author contributions

XW, BF, and CZ are responsible for formulating overarching research goals and aims. PZ, RZ, and RC are accountable for the application of statistics. CZ and XC are responsible for verifying the overall replication of results and other research outputs. XC and PZ are responsible for explicitly writing the initial draught. Y-HJ, FH, SL, and LL are responsible for critical review, commentary, or revision—including pre- or post-publication stages. XW, BF, CZ, and LC are responsible for supervision, oversight, and leadership responsibility for the research activity planning and execution, including mentorship external to the core team. All authors contributed to the article and approved the submitted version.

## Footnotes

^1^Health care-acquired infections (HAIs).

^2^Infection prevention and control (IPC).

^3^World Health Organization (WHO).

^4^Environmental service workers (ESWs).

^5^Knowledge, Attitudes, Practise, and Experience regarding Infection Prevention and Control-associated Questionnaire (KAPE-IPC-Q).
